# Modeling of Fiber-Constrained Planar PVC Gel Actuators

**DOI:** 10.3390/nano13091483

**Published:** 2023-04-26

**Authors:** Yi Li, Xuxin Feng, Lixiang Zhu, Ziqian Zhang, Mingfei Guo, Zhixin Li, Yanbiao Li, Minoru Hashimoto

**Affiliations:** 1 Key Laboratory of Special Purpose Equipment and Advanced Processing Technology of Ministry of Education, Zhejiang University of Technology, Hangzhou 310032, Chinalybrory@zjut.edu.cn (Y.L.); 2 Zhejiang Provincial Key Laboratory of Special Purpose Equipment and Advanced Processing Technology, Zhejiang University of Technology, Hangzhou 310032, China; 3 Faculty of Textile Science and Technology, Shinshu University, 3-15-1 Tokida, Ueda 386-8567, Japan; hashi@shinshu-u.ac.jp

**Keywords:** PVC gel, fiber-constrained, planar actuator, EAP

## Abstract

In recent years, plasticized poly (vinyl chloride) (PVC) gel has attracted increasing attention in soft robotics. However, there is scarce research on the deformation mechanism and modeling of PVC gel actuators. In this study, to investigate the deformation mechanism of fiber-constrained planar PVC gel actuators, we propose a complex nonlinear model based on traditional thermodynamic electroactive polymer (EAP) multi-field coupling theory. The proposed model can reveal the dielectric breakdown strength of PVC gels and predict the deformation of planar PVC gel actuators with varying levels of pre-stretching. The theoretical results were in good agreement with the experimental results, indicating the feasibility of the proposed model.

## 1. Introduction

Over the past two decades, EAPs have attracted considerable attention from researchers and engineers owing to their exceptional characteristics, such as high compliance with large actuation strain and stress and ease of fabrication and processing [1,2,3]. These properties allow EAPs to be used in various advanced applications, such as soft sensors and actuators, flexible electronics, and bio-inspired soft robotics [4,5,6,7,8,9]. Dielectric elastomers are prevalent dielectric EAP because of their non-conducting nature and relatively high rigidity, which require a high driving voltage. Upon the application of voltage, a dielectric elastomer membrane expands its area, exhibiting an electrodynamic strain of more than 100% [10]. The PVC gel, a composite dielectric gel, is another example of a dielectric EAP formed by the diffusion of small molecules into polymer networks. Owing to its high internal solvent content, the gel was softer than the elastomer, thereby producing significant deformation under low driving voltage. Under the different boundary conditions of the gel membrane and electrode [11], the PVC gel exhibited substantial electrically induced amoeba-like creep deformation, which can induce various movements. The plasticized multilayer PVC gel actuator with meshed electrodes demonstrated performance comparable to that of human muscles at a low driving voltage (<0.4 kV) [12]. Thus, it is a promising candidate for realizing practical soft robots and devices. It has been used in various applications, such as optical focusing lenses [13], strain sensors [14], walking assistance wearables [15], wearable sensing devices [16,17], nanogenerators [18], biomimetic jellyfish, adhesives, and fishtails [19,20,21].

Although significant progress has been achieved in the development of PVC gel-based devices, few studies have focused on modeling the deformation of PVC gels and their actuators. Hirai, T., et al. [22] explained the creep deformation mechanism of PVC gel using the variation of space charge density in solvent-rich layer. On this basis, Asaka, K., et al. [23] developed an electro-mechanical model on the basis of the electrochemical properties of PVC gel, which effectively estimated the bending displacement of the actuator at a given voltage. The effect of ionic liquid on improving the actuator performance was clearly described by this model in a later study [24]. On the other hand, Frank, Z., et al. [25] used finite element method to simulate the deformation of PVC gel actuators with meshed electrodes. In addition, Al-Rubaiai, M., et al. [26] used a data-driven approach to model the nonlinear dynamics of the PVC gel actuator. A Hammerstein model composed of a nonlinear module cascaded with linear dynamics was proposed to describe the dependence of the voltage input displacement—output frequency response on the input voltage amplitude and bias. However, most of these models are limited to specific actuator types and are phenomenological or empirical. In the field of soft robotics, accurate predictive models are necessary for the implementation and control of these actuators, which can be achieved using numerical computational models with real physical significance.

The electrical deformation of the PVC gel actuator is highly complex and non-linear [11,27,28], depending on various factors, such as gel composition, voltage amplitude, and electrode configuration. In this study, a numerical model based on thermodynamics is proposed for the theoretical deformation of PVC gel actuators with a fiber-constrained structure. The material parameters of the PVC gels were determined by experimental measurements, and the boundary conditions of the proposed fiber-constrained planar actuator were determined by theoretical analysis. The results demonstrate that the proposed model can effectively predict the electrical response of planar PVC gel actuators under varying horizontal pre-stretching and voltage amplitudes, which will facilitate the design and tuning of PVC gel actuators. Notably, the proposed model reveals the existence of complex phenomena in PVC gels, such as electrically induced independent polarization of PVC and DBA, electrical breakdown, and tension loss conditions, which have not been comprehensively explored in previous models.

## 2. Methodology

### 2.1. Modeling of PVC Gel Deformation

**Figure 1:**
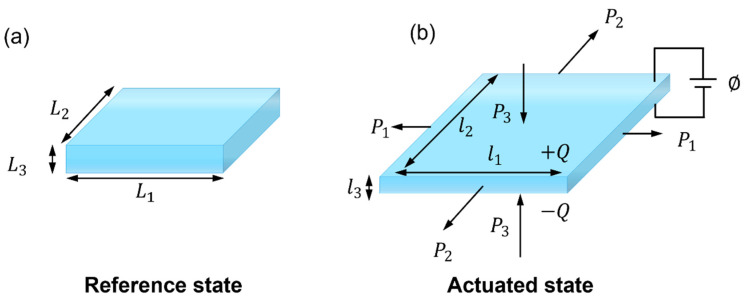
Deformation of PVC gel membrane (**a**) Reference. (**b**) Actuated state.

Herein, we summarize the deformation theory of PVC gels related to this study. The PVC gel is deformed by the applied force and voltage (Figure 1). In the reference state, without force and voltage, the thickness, length, and width of the membrane are $L_{3}$, $L_{1}$, and $L_{2}$, respectively. In the actuated deformation state, the applied forces are $P_{1}$, $P_{2}$, and $P_{3}$, the applied voltage is ϕ, thickness of the membrane is $l_{3}$, length is $l_{1}$, and the width is $l_{2}$. The thermodynamic equilibrium equation is as follows:

(1) $$\delta F=P_{1}\delta l_{1}+P_{2}\delta l_{2}+P_{3}\delta l_{3}+\phi \delta Q$$

where δF is the increment of the system free energy, $\delta l_{1}$, $\delta l_{2}$, and $\delta l_{3}$ are the dimensional changes in each direction, $P_{1}\delta l_{1}+P_{2}\delta l_{2}+P_{3}\delta l_{3}$ is the work done by the mechanical energy, and ϕδQ is the work done by the electric field. When the dielectric force and voltage are balanced, the increment in free energy is equal to the work done by the force and voltage. Here, we did not consider the work done by the chemical potential energy formed by the swelling of dielectric gel as Li Bo’s derivation [10] because the preparation method of PVC gel was made through the gelation of polymer mixed solution and did not consider the part of the Helmholtz free energy caused by the extension of the polymer network caused by the swelling of the crosslinked polymer body. The initial state of our model is the gel state after the aggregation network is extended.

$W=F/(L_{1}L_{2}L_{3})$ is defined as a specific Helmholtz free energy density; $\lambda_{1}=l_{1}/L_{1}$, $\lambda_{2}=l_{2}/L_{2}$, and $\lambda_{3}=l_{3}/L_{3}$ are the driven deformations in three directions; $\sigma_{1}=P_{1}/l_{2}l_{3}$, $\sigma_{2}=P_{2}/l_{1}l_{3}$, and $\sigma_{3}=P_{3}/l_{1}l_{2}$ are the real stresses in three directions; $E=\phi /l_{3}$ is the true electric field intensity; $D=Q/l_{1}l_{2}$ is the true electric displacement; and the charge quantity $Q=Dl_{1}l_{2}$ on the electrode at any point is related to the electric displacement, so the change in charge is:

(2) $$\delta Q=D\delta l_{1}l_{2}+Dl_{1}\delta l_{2}+l_{1}l_{2}\delta D$$

Our material was determined to be incompressible; therefore, $\lambda_{1}\lambda_{2}\lambda_{3}=1$, and the deformation in either direction was determined by the deformation in the other two directions. Therefore, $\delta \lambda_{1}$ and $\delta \lambda_{2}$ represent $\delta \lambda_{3}$ as:

(3) $$\delta \lambda_{3}=-\frac{\delta \lambda_{1}}{\lambda_{1}^{2}\lambda_{2}}-\frac{\delta \lambda_{2}}{\lambda_{2}^{2}\lambda_{1}}$$

At the same time, divide both sides of Equation (1) by the volume $L_{1}L_{2}L_{3}$ of PVC gel, and then substitute Equations (2) and (3) into the equation to obtain:

(4) $$\delta W=\frac{\sigma_{1}-\sigma_{3}+DE}{\lambda_{1}}\delta \lambda_{1}+\frac{\sigma_{2}-\sigma_{3}+DE}{\lambda_{2}}\delta \lambda_{2}+E\delta D$$

On the basis of the incompressibility theory of the material model, (4) applies to arbitrary and independent changes $\delta \lambda_{1}$, $\delta \lambda_{2}$, and δD. In the material model, we define the free energy $W=W(\lambda_{1},\lambda_{2},D)$ as a function of three independent variables, so (4) can be changed to:

(5) $$\left(\frac{\partial W}{\partial \lambda_{1}}-\frac{\sigma_{1}-\sigma_{3}+DE}{\lambda_{1}}\right)\delta \lambda_{1}+\left(\frac{\partial W}{\partial \lambda_{2}}-\frac{\sigma_{2}-\sigma_{3}+DE}{\lambda_{2}}\right)\delta \lambda_{2}+\left(\frac{\partial W}{\partial D}-E\right)\delta D=0$$

Meanwhile, (5) is equivalent to:

(6) $$\sigma_{1}-\sigma_{3}=\lambda_{1}\frac{\partial W(\lambda_{1},\lambda_{2},D)}{\partial \lambda_{1}}-DE$$

(7) $$\sigma_{2}-\sigma_{3}=\lambda_{2}\frac{\partial W(\lambda_{1},\lambda_{2},D)}{\partial \lambda_{2}}-DE$$

(8) $$E=\frac{\partial W(\lambda_{1},\lambda_{2},D)}{\partial D}$$

As an idealized model, it is assumed that the real electric field in the dielectric behavior of the PVC gel is related to the real electric displacement as follows:

(9) E=D/ε

where ε is the dielectric constant of the PVC gel, which is assumed to be a constant unrelated to the electric field strength and deformation. Substituting (9) into (4) and integrating δD yields:

(10) $$W\left(\lambda_{1},\lambda_{2},D\right)=W_{S}(\lambda_{1},\lambda_{2})+\frac{D^{2}}{2\varepsilon }$$

where $W_{S}(\lambda_{1},\lambda_{2})$ is the Helmholtz free energy integral constant related to PVC gel stretching, and the material is incompressible. Therefore, the model is an ideal PVC gel deformation model, in which mechanical stretching and electric field polarization independently contribute to the free energy. Therefore, (10) is substituted into (6) and (7) to obtain the following formulas:

(11) $$\sigma_{1}-\sigma_{3}=\lambda_{1}\frac{\partial W_{S}(\lambda_{1},\lambda_{2})}{\partial \lambda_{1}}-\varepsilon E^{2}$$

(12) $$\sigma_{2}-\sigma_{3}=\lambda_{2}\frac{\partial W_{S}(\lambda_{1},\lambda_{2})}{\partial \lambda_{2}}-\varepsilon E^{2}$$

The deformation free energy of the electromechanical coupling effect can be characterized by two parts: the strain energy $W_{stretching}$ generated by mechanical tension and free energy $W_{polarizing}$ generated by electric field polarization:

(13) $$W=W_{stretching}(\lambda_{1},\lambda_{2})+W_{polarizing}(D)$$

Here, the two free energy expressions are given in the form of the Gent model [29] and static energy:

(14) $$W_{stretching}\left(\lambda_{1},\lambda_{2}\right)=-\frac{\mu J_{lim}}{2}\log(1-\frac{\lambda_{1}^{2}+\lambda_{2}^{2}+\lambda_{1}^{-2}\lambda_{2}^{-2}-3}{J_{lim}})$$

(15) $$W_{polarizing}\left(D\right)=\int_{0}^{D}EdD=\int_{0}^{D}f(D)dD$$

where μ is the shear modulus of the PVC gel and $J_{lim}$ is the ultimate tensile correlation coefficient [30]. When the system has a small deformation, $\lambda_{1}^{2}+\lambda_{2}^{2}+\lambda_{1}^{-2}\lambda_{2}^{-2}-3\ll J_{lim}$, the Gent model reverts to the neo-Hookean model [31] as follows:

(16) $$W_{stretching}\left(\lambda_{1},\lambda_{2}\right)=\frac{\mu }{2}(\lambda_{1}^{2}+\lambda_{2}^{2}+\lambda_{1}^{-2}\lambda_{2}^{-2}-3)$$

As the tensile deformation approached the limit of $\lambda_{1}^{2}+\lambda_{2}^{2}+\lambda_{1}^{-2}\lambda_{2}^{-2}-3\to J_{lim}$, the Gent models hardened sharply, conforming to the mechanical properties of the PVC gel.

The polarizations of the polymer elastomer and the solvent in the PVC gel were defined separately. The PVC polymer molecule follows linear polarization, whereas the DBA solvent molecule reaches the polarization saturation state, dividing the polarization free energy $W_{polarizing}(D)$ into two parts. The nonuniform deformation of the PVC gel can be represented as:

(17) $$W_{polarizing}\left(D\right)=W_{m_{polarizing}}\left(D\right)+W_{s_{polarizing}}\left(D\right)=(1-\eta )\int_{0}^{D_{m}}EdD_{m}+\eta \int_{0}^{D_{s}}EdD_{s}$$

where $W_{m\_polarizing}(D)$ is the free energy generated by the molecular polarization of the PVC polymer, $W_{s\_polarizing}(D)$ is the free energy generated by the molecular polarization of the DBA solvent, and η is the volume fraction of the DBA solvent.

By substituting (14) and (17) into (11) and (12), the state equilibrium equation of the PVC gel is obtained as follows:

(18) $$\sigma_{1}+(1-\eta )\varepsilon_{m}E^{2}+\eta \bar{D}\tan h(\varepsilon_{s}E/\bar{D})E=\frac{\mu (\lambda_{1}^{2}-\lambda_{1}^{-2}\lambda_{2}^{-2})}{1-(\lambda_{1}^{2}+\lambda_{2}^{2}+\lambda_{1}^{-2}\lambda_{2}^{-2}-3)/J_{lim}}$$

(19) $$\sigma_{2}+(1-\eta )\varepsilon_{m}E^{2}+\eta \bar{D}\tan h(\varepsilon_{s}E/\bar{D})E=\frac{\mu (\lambda_{2}^{2}-\lambda_{1}^{-2}\lambda_{2}^{-2})}{1-(\lambda_{1}^{2}+\lambda_{2}^{2}+\lambda_{1}^{-2}\lambda_{2}^{-2}-3)/J_{lim}}$$

where η is the volume fraction of the solvent, $\varepsilon_{m}$ is the permittivity of the polymer, $\varepsilon_{s}$ is the permittivity of the solvent, and $\bar{D}$ is the polarization-limiting constant. Here, the hyperbolic tangent function was used to describe the saturation polarization of the DBA solvent molecule.

### 2.2. The Model of the Proposed Planar Actuator

**Figure 2:**
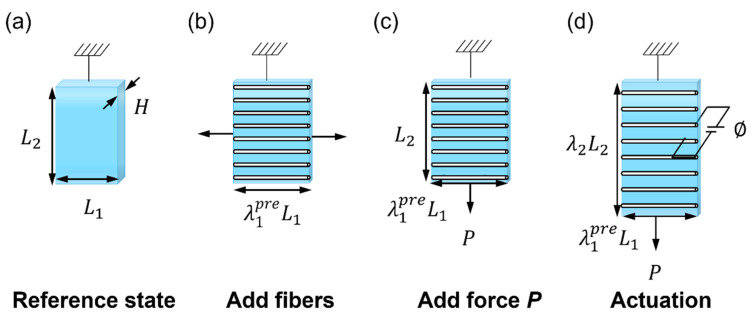
The fiber-constrained planar PVC gel planar actuator. (**a**) In the reference state, the membrane is not stretched. (**b**) The membrane is pre-stretched in the horizontal direction, and fibers are added. (**c**) A force P is applied in the vertical direction so that the vertical direction returns to its original length $L_{2}$. (**d**) The membrane is subjected to both force P and voltage ϕ.

Using the above PVC gel deformation model, the deformation of different PVC gel actuator structures under a specified force and voltage can be theoretically calculated. The actuator structure proposed in this paper is shown in Figure 2a. In the reference state, the membrane is not affected by force and voltage, and the thickness, length, and width of the PVC gel are H, $L_{1}$, and $L_{2}$ respectively; (b) The membrane is then stretched horizontally, and the fibers are uniformly added horizontally. By adjusting the mechanical force, the pre-stretch $\lambda_{1}^{pre}$ in the horizontal direction could be adjusted over a wide range; (c) When a mechanical force is released, the horizontal length of the membrane is fixed by the fiber at $\lambda_{1}^{pre}L_{1}$. Next, a force P is applied to the membrane in the vertical direction so that the vertical dimension is restored to the initial state $L_{2}$, so that the vertical pre-stretch $\lambda_{2}^{pre}=1$; (d) In the driving state, the membrane is subjected to both force P and voltage ϕ using flexible electrodes. The horizontal deformation is maintained as $\lambda_{1}=\lambda_{1}^{pre}$, and the vertical deformation is $\lambda_{2}$.

According to the structure of the PVC gel actuator, boundary conditions are specified for the states (18) and (19): the PVC gel membrane may not be fully constrained by the fiber. When the horizontal stress in the membrane is stretched, the fiber is in a state of compression, and the fiber may bend. Conversely, when the fiber is stretched, the horizontal stress within the membrane is compressed. It is assumed that the membrane cannot withstand the compressive stress because it will cause the membrane to fold; that is, the horizontal stretch is completely constrained, so that fiber bending and membrane folding will not occur. $\sigma_{1}=0$ is the condition of tension loss, under which is the allowable drive, $\sigma_{2}$ is determined by P, $\sigma_{3}=0$, and $\lambda_{1}=\lambda_{1}^{pre}$; each parameter is substituted into Formulas (18) and (19), and the equation of state of the actuator is:

(20) $$\left(1-\eta )\varepsilon_{m}(\lambda_{1}^{pre}\lambda_{2}\frac{\phi }{H}{)}^{2}+\eta (\lambda_{1}^{pre}\lambda_{2}\frac{\phi }{H})\bar{D}\tan h(\varepsilon_{s}\lambda_{1}^{pre}\lambda_{2}\frac{\phi }{\bar{D}H}\right)\;=\frac{\mu ((\lambda_{1}^{pre}{)}^{2}-(\lambda_{1}^{pre}\lambda_{2}{)}^{-2})}{1-((\lambda_{1}^{pre}{)}^{2}+\lambda_{2}^{2}+(\lambda_{1}^{pre}\lambda_{2}{)}^{-2}-3)/J_{lim}}$$

(21) $$\lambda_{2}\frac{P}{L_{1}H}+(1-\eta )\varepsilon_{m}(\lambda_{1}^{pre}\lambda_{2}\frac{\phi }{H}{)}^{2}+\eta (\lambda_{1}^{pre}\lambda_{2}\frac{\phi }{H})\bar{D}\tan h(\varepsilon_{s}\lambda_{1}^{pre}\lambda_{2}\frac{\phi }{\bar{D}H})\;=\frac{\mu ({\lambda_{2}}^{2}-(\lambda_{1}^{pre}\lambda_{2}{)}^{-2})}{1-((\lambda_{1}^{pre}{)}^{2}+\lambda_{2}^{2}+(\lambda_{1}^{pre}\lambda_{2}{)}^{-2}-3)/J_{lim}}$$

### 2.3. The Amoeba-like Deformation Mechanism of PVC Gels

**Figure 3:**
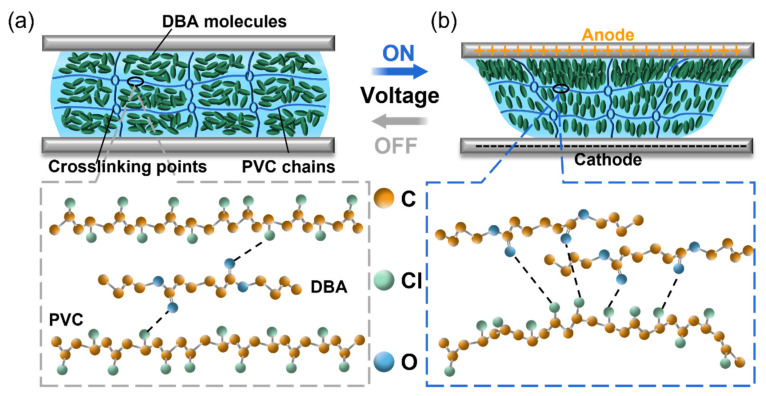
Internal structure of PVC gel. (**a**) Before the voltage was applied, DBA was evenly dissolved inside the gel. (**b**) After the voltage is applied, the DBA migrates to the anode, resulting in creep deformation owing to interaction with the PVC chains.

**Figure 4:**
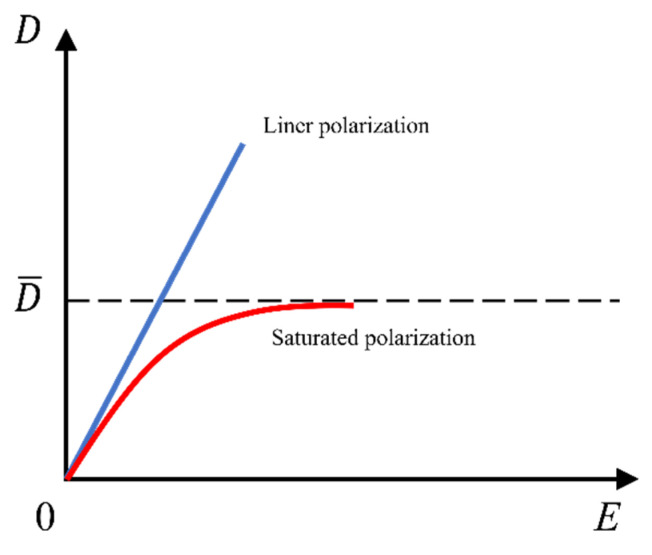
Two types of polarization in PVC gel.

Here, we make an important distinction between the deformation theory of PVC gels and that of dielectric elastomers. Ali, M., et al. [27] studied and elaborated on the electrode-formation-induced structure of PVC gel (Figure 3). Before the energized field was applied, each PVC chain was loosely connected through physical crosslinking points, and the PVC internal space was filled with Dibutyl adipate (DBA). The dipole rotations of DBA and PVC are random and in an equilibrium state. Under the action of voltage, the dipoles of DBA and PVC migrate to the anode side until saturation because the dipoles of PVC and DBA interact with each other. With the enrichment of DBA on the anode side, the PVC chain segments and crosslinking points were pulled to the anode side, resulting in amoeba-like creep deformation. We describe this phenomenon in terms of the independent polarization of the polymer and solvent molecules. Figure 4 shows the two types of polarization in the PVC gel. PVC polymer molecules follow linear polarization, DBA solvent molecules follow saturated polarization, and the saturation function uses the hyperbolic tangent function.

## 3. Experimental Section

### 3.1. Materials

PVC power (CAS 9002-86-2), plasticizer DBA (CAS 105997, 96%), and a solution (Tetrahydrofuran (THF), CAS 109,999, 99.9%) were purchased from Sigma-Aldrich. The flexible electrode material (Oil compound KS-660) was purchased from Shin-Etsu Chemical. All the chemicals were used without further purification.

### 3.2. Preparation of PVC Gel Membrane

**Figure 5:**
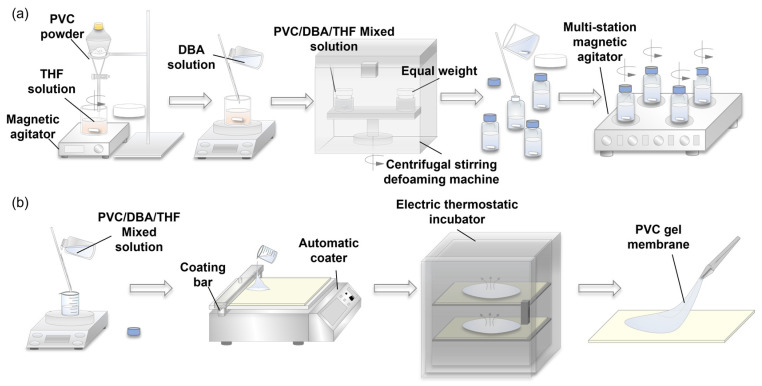
Preparation of PVC gel membranes: (**a**) preparation of PVC/DBA/THF mixed solution and (**b**) preparation of PVC gel molding by coating method.

The PVC gel was prepared by the direct gelation of a PVC/DBA/THF mixed solution. The preparation process of the mixed solution is shown in Figure 5a. The mixed solution was centrifuged for 6 min at 3000 rpm. The final mixed solution was stirred at 1500 rpm (room temperature) for 24 h before use. In this study, PVC gel membranes were prepared using a coating method, as shown in Figure 5b. The mixed solution was cast on a glass slide and deformed using an applicator roll. PVC gel membranes were obtained after THF evaporation at a constant temperature of approximately 20 °C for two days. The weight ratio of PVC to DBA was 1:6, and the thickness of the membrane was approximately 0.2 mm.

### 3.3. Preparation of Fiber-Constrained Planar PVC Gel Actuators

**Figure 6:**
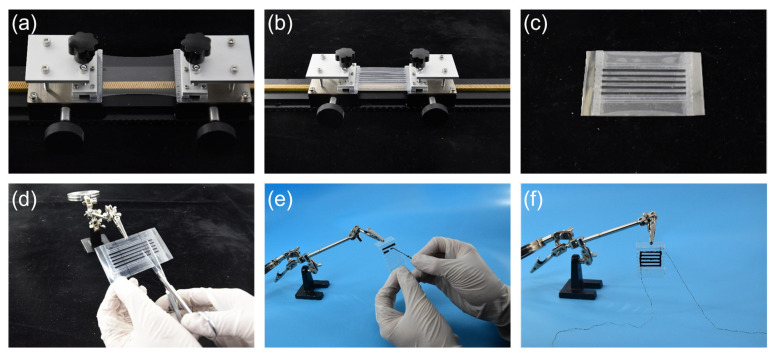
The preparation of the fiber-constrained planar PVC gel actuator: (**a**) PVC gel membrane was clamped on the self-designed tensile mechanism for uniaxial stretching; (**b**) fibers were used for restraint on the first side of the membrane; (**c**) fibers were used for restraint on the opposite side of the membrane; (**d**) the clamping part was cut off, and the driving direction of the actuator was released; (**e**) both sides of the planar actuator were coated with silicone grease electrodes; and (**f**) external wires were connected on both sides to complete the overall preparation of the planar actuator.

We used fibers to uniaxially constrain the bending deformation of the PVC gel membranes to develop a biological muscle-like unidirectional actuation planar PVC gel actuator. In addition, a unidimensional pre-stretch was applied to the PVC gel membrane in the non-actuation direction, resulting in easier actuation in the actuation direction. PET adhesive sheets with a thickness of 0.2 mm were used as fibers. Unidirectional pre-stretching of 0–300% was applied to the membranes, and conductive silicone grease (KS-660) was used as the flexible electrode. A fiber-constrained planar PVC gel actuator was prepared as shown in Figure 6.

### 3.4. Experimental Set

**Figure 7:**
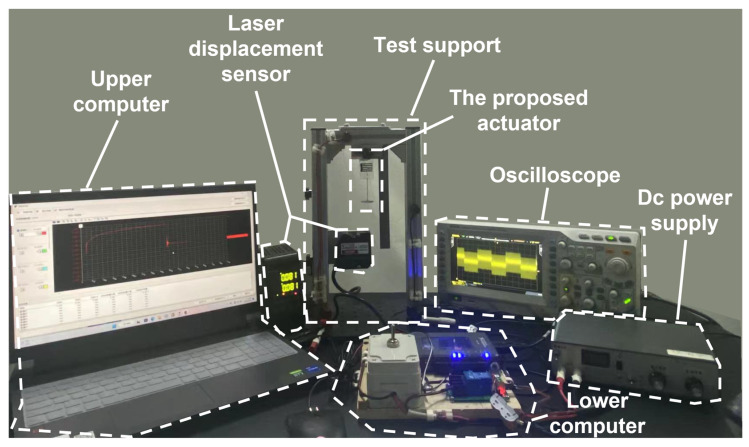
The proposed PVC gel planar actuator test platform and equipment used.

The test platform of the proposed planar actuator and the test equipment used are shown in Figure 7. The MyRIO software (National Instruments) in the upper computer was used to control the on/off of the DC power supply so that the actuator received DC square wave signals of the specified frequency. A series resistance in the circuit was used to monitor the current signal with an oscilloscope (RIGOL DS2102A) for the electric breakdown warning of the actuator. The planar actuator was clamped on a self-designed test bracket, connected to the control circuit of the lower computer, and a laser displacement sensor (KEYENCE LK-H050) was installed under the bracket to measure the displacement in the vertical direction of the planar actuator.

## 4. Results and Discussion

**Figure 8:**
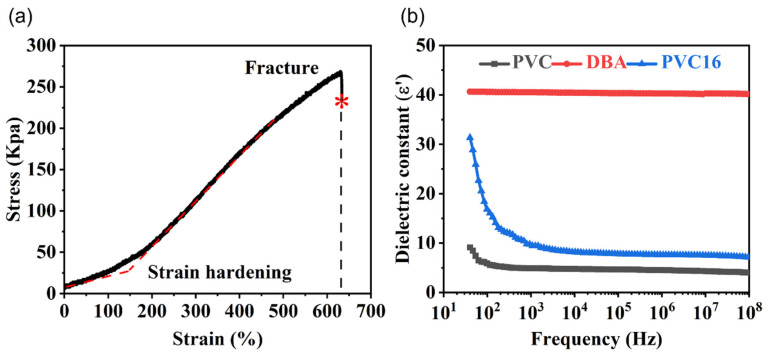
Experimental results of PVC16 gel membranes. (**a**) Uniaxial tensile test results: stress–strain curves. The asterisk is the uniaxial tensile fracture point of PVC gel. The red dashed line shows the change of elastic modulus of PVC gel. (**b**) Dielectric constants of PVC, DBA, and PVC16 as functions of the frequency.

**Figure 9:**
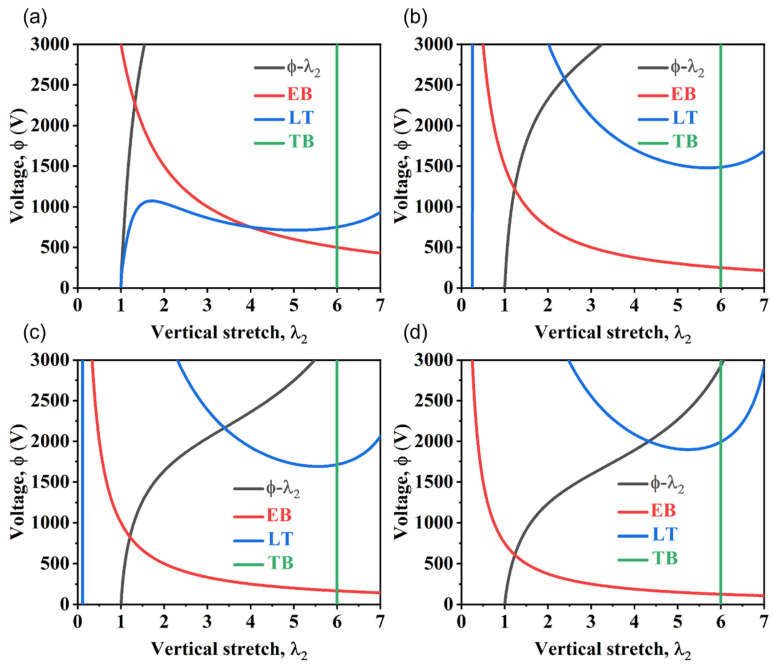
Theoretical deformation of the proposed actuator under different horizontal pre-stretches: (**a**) $\lambda_{1}^{pre}=0$, (**b**) $\lambda_{1}^{pre}=1$, (**c**) $\lambda_{1}^{pre}=2$, and (**d**) $\lambda_{1}^{pre}=3$.

According to the uniaxial tensile test (Figure 8a), the shear modulus of the PVC gel was μ=7 kPa, the mechanical tensile limit was $\lambda_{lim}=6$, and the material constant related to the ultimate tensile strength was $J_{lim}=2\lambda_{lim}^{2}+\lambda_{lim}^{-4}-3=69$. According to the dielectric constant test (Figure 8b), in the high-frequency stable region, the relative dielectric constant of PVC was $\varepsilon_{m}^{\prime }=5$ and that of the DBA was $\varepsilon_{s}^{\prime }=40$. The vacuum permittivity $\varepsilon_{0}=8.854187817\times 10^{-12}$ Fm^−1^. The permittivity of PVC polymer was $\varepsilon_{m}=\varepsilon_{m}^{\prime }\varepsilon_{0}=4.427\times 10^{-11}$ Fm^−1^, and that of DBA solvent was $\varepsilon_{s}=\varepsilon_{s}^{\prime }\varepsilon_{0}=3.542\times 10^{-10}$ Fm^−1^. According to the relevant literature [10], the polarization limit was set to $\bar{D}=100\sqrt{\varepsilon_{s}NKT}$, where K is the Boltzmann constant, T is the temperature, and N is the number of crosslinks per unit of initial volume, which can be obtained by rubber elasticity theory: N=μ/(KT). According to the above calculation method, the variation of $\bar{D}$ in the range of 8 × 10^−5^ to 5.3 × 10^−4^ was calculated to fit the experimental data. The volume fraction of DBA solvent was approximately η=6/7. The thickness, width, and length of the PVC gel in the reference state were set as H=200 μm, $L_{1}=25$ mm, and $L_{2}=20$ mm, respectively. The constant breakdown electric field of the PVC gel was set as $E_{EB}=15$ V/μm [29]. Finally, the original length in the vertical direction was restored according to the elasticity of the PVC gel. Equation (21) is drawn as a voltage–stretch curve ($\phi -\lambda_{2}$), and (20) is drawn as a condition of tension loss (LT) in Figure 9, where the electric field breakdown curve is drawn as EB, and the mechanical tensile fracture curve is drawn as TB.

According to the proposed model, we can explore the deformation mechanism of planar actuators under different horizontal pre-stretches and reveal the allowable range, electrical breakdown limit, and mechanical failure limit of the actuators. Under different horizontal pre-stretches (Figure 9), the black curve ($\phi -\lambda_{2}$) represents the actuator deformation curve predicted by the model, which can predict the strain value of the actuator under different voltages. The red curve (EB) represents the electrical breakdown strength of PVC gel. The blue curve (LT) represents the tension loss condition and the allowable range of the actuator under the blue curve. In theory, the intersection points of the black and red curves represent the maximum strain that can be obtained by the actuators. Maximum strains of 31.5%, 22.5%, 20.7%, and 25% can be generated by the four types of actuators with different horizontal pre-stretches at 2280, 1224, 816, and 600 V, respectively. In general, with the increase in horizontal pre-stretching, planar actuators can achieve relatively large deformations under lower voltages, which is the key to improving the performance of planar actuators. However, as the horizontal pre-stretch increases, the PVC gel becomes thinner, so electrical breakdown will occur at lower voltages, and the conditions for horizontal tension loss will expand with the increase in pre-stretching. The mechanical failure limit is defined as the uniaxial tensile limit fracture rate (d) and, therefore, is set to a constant value in the vertical direction.

**Figure 10:**
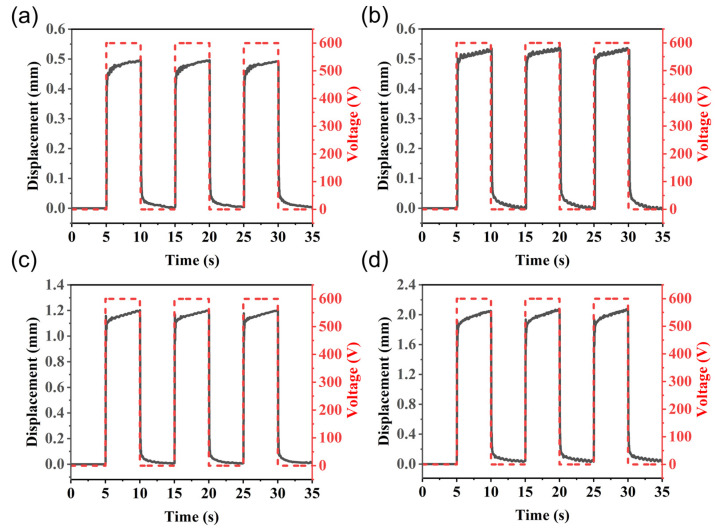
Displacement of four different horizontally pre-stretched planar actuators at 600 V, 0.1 Hz: (**a**) $\lambda_{1}^{pre}=0$, (**b**) $\lambda_{1}^{pre}=1$, (**c**) $\lambda_{1}^{pre}=2$, and (**d**) $\lambda_{1}^{pre}=3$.

**Figure 11:**
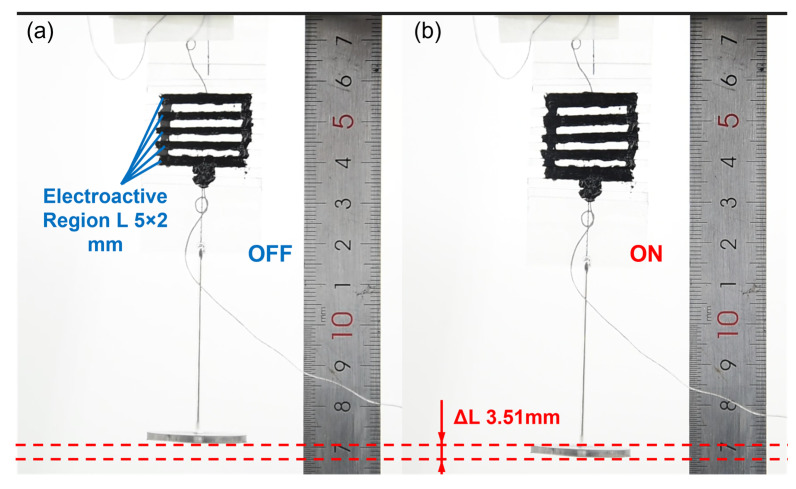
Deformation of a 200% horizontal pre-stretch planar actuator at 1100 V: (**a**) before the voltage was applied and (**b**) after the voltage was applied.

In this experiment, four types of planar actuators were tested under a horizontal pre-stretch, including horizontal pre-stretch of 0%, 100%, 200%, and 300%. Each horizontal pre-stretch consisted of five samples. The DC voltage signal was controlled at a frequency of 0.1 Hz, and three displacement data were recorded by a laser displacement sensor. Figure 10 shows the displacement for the four types of actuators at 0.1 Hz and 600 V. In theory, the width of the fiber can be very small, which is also ignored in the above model. However, in reality, it is difficult to find a sufficiently thin fiber to fully constrain the pre-stretched gel membrane. Therefore, the actual experimental test was based on the electroactive region of the planar actuator, namely the gel region coated with the black electrode as in Figure 11, and the original driving length is 5 × 2 mm. At a voltage of 1100 V, a planar actuator with a 200% horizontal pre-stretch provided a displacement of 3.51 mm in the vertical direction, thus achieving a 35.1% strain, which is very close to the 36.4% theoretical value of the mathematical model.

**Figure 12:**
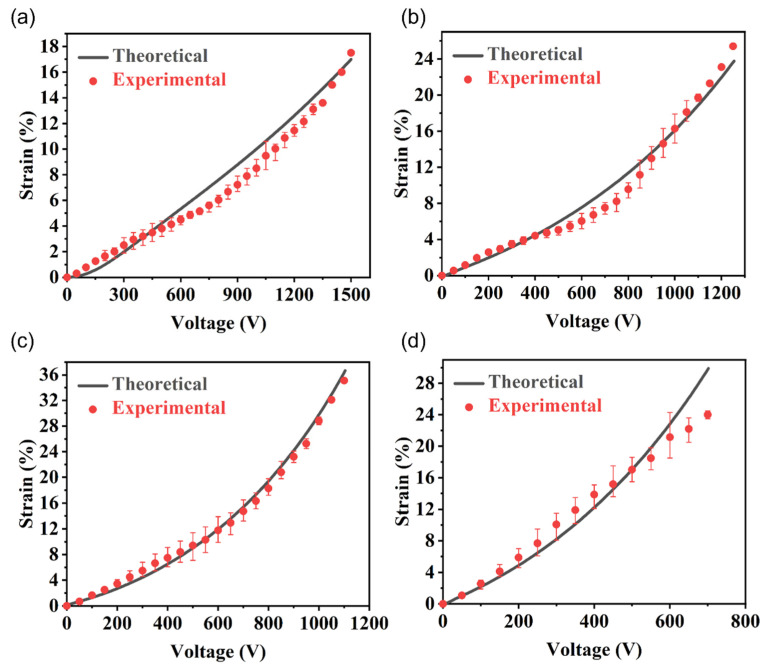
Comparison of theoretical deformation and experimental results of fiber-constrained planar PVC gel actuators under different horizontal pre-stretches. Horizontal pre-stretch: (**a**) $\lambda_{1}^{pre}=0$, (**b**) $\lambda_{1}^{pre}=1$, (**c**) $\lambda_{1}^{pre}=2$, and (**d**) $\lambda_{1}^{pre}=3$.

Because the electric breakdown field strength set by our theoretical model is a fixed value, it can only preliminarily predict the deformation of fiber-constrained planar actuators in the general range. However, in the actual testing process, we found that the electrical breakdown strength differed under different horizontal pre-stretches. We substituted the electrical breakdown strength measured in the experiment and the deformation data before the electrical breakdown into the new model and performed parameter fitting of the model, as shown in Figure 12. The polarization limits $\bar{D}$ set by theoretical deformation under 0%, 100%, 200%, and 300% horizontal pre-stretch were 5.3 × 10^−4^, 1.3 × 10^−4^, 8 × 10^−5^, and 1.2 × 10^−4^, respectively. The breakdown voltages of 0%, 100%, 200%, and 300% horizontal pre-stretch were 1500, 1200, 1100, and 700 V, respectively. The theoretical and experimental deformations corresponding to the vertical direction were 17.0, 23.6, 36.4, 29.9, 17.5, 25.4, 35.1, and 24%, respectively. The theoretical results are in good agreement with the experimental results. Moreover, it can be seen that the variation trend of the theoretical curve is consistent with that of the actual test data. The higher the voltage growth, the faster the deformation growth. The new model and the overall theory are in good agreement with the actual situation. As shown in Figure 12c, the theory of a 200% horizontal pre-stretch was the most consistent with the experiment. This is because the Gent model and hyperbolic tangent function are used in our model mainly to characterize the nonlinearity of the PVC gel deformation. The Gent model will enhance the nonlinearity with the increase of gel deformation; that is, the larger the pre-stretching amount, the stronger the nonlinearity. The 200% horizontal pre-stretch group could measure the most complete experimental data, and the breakdown value measured in the experiment was the closest to the predicted breakdown value of the original model. Therefore, when parameter fitting was performed, the fitting value was the best, and the trend linearity was the most consistent. The relative nonlinear degree of the driving curve before breakdown predicted by the original model for the 0% and 100% groups was low, and the fitting with the nonlinear data measured by the experiment was poor. In addition, the samples of the 300% group broke down very early in the experimental test, and the breakdown intensity was far lower than the fixed value set by the model and, therefore, did not fit well.

## 5. Conclusions

We developed a theoretical deformation model for PVC gels using the EAP multi-field coupling theory based on conventional thermodynamics. In this model, the specific amoeba-like creep deformation behavior of the PVC gel is explained by the different polarizations of the PVC gel polymer and solvent molecules, and the free energy function is established by testing the mechanical and dielectric properties of the material, thus enabling the simulation of the complex nonlinear electrochemical–mechanical behavior of PVC gels. This was applied to the modelling of the fiber-constrained planar PVC gel actuator. Through a combination of experimental tests and numerical simulations, it was found that theory and practice were in good agreement. Thus, the feasibility of the proposed model is verified. We expect our theoretical deformation model of PVC gel to serve as an inspiration for the theoretical structural design of other PVC gel-based devices and expect that PVC gel actuators will be used in the near future in areas such as soft robotics. 

## Data Availability

Not applicable.
